# Lectures on Obstetric Nursing

**Published:** 1889-11

**Authors:** 


**Lectures on Obstetric Nursing, delivered at the Training School for Nurses of the Philadelphia Hospital.** By THEOPHILUS PARVIN, M. D., Professor of Obstetrics and Diseases of Women and Children, at Jefferson Medical College. Philadelphia: P. Blakiston, Son & Co., 1012 Walnut street. 1889. Buffalo: Peter Paul & Brother. Price, 75 cents.

This little work contains the practical experience of one of the best American teachers on the subjects of which it treats, and, like all of the author's writings on the science and art of obstetrics, is clear and practical, and clothed in the choicest language. It is full of excellent advice, the result of his long and successful practice, to nurses on the duties devolving upon them in the management of the patient during labor, of their responsibilities, and especially of the loyalty they owe to the accoucheur. The second lecture treats briefly of Sepsis and Antiseptics, the preparation of the bed, the sanitary condition of the lying-in-room, etc. We find so much to endorse in the work that we wish it could be placed in the hand of every nurse, who cares for parturient and puerperal women, and who is zealous for wise counsel in her responsible position.

